# Mediating Role of Loneliness on Stigma and Depressive Symptoms Among Older Persons Living With HIV

**DOI:** 10.1093/geroni/igab046.666

**Published:** 2021-12-17

**Authors:** Moka Yoo-Jeong

**Affiliations:** Northeastern University, Brookline, Massachusetts, United States

## Abstract

Studies have shown associations among stigma, loneliness, and depressive symptoms in older persons living with HIV (OPLWH) but research assessing the mediating pathway among these variables is lacking. As such, the aims of this study were to assess the association between stigma and depressive symptoms and to test the mediating effects of loneliness. A sample of 146 OPLWH (50 years of age and older) recruited from an outpatient HIV clinic in Atlanta, GA, completed a cross-sectional survey. Mediation analysis, guided by Baron and Kenny’s (1986) criteria, was conducted using Stata v14.2 to assess the direct and indirect effects of loneliness on the association between stigma and depressive symptoms while controlling for covariates (self-rated health [0=poor to fair, 1=good to excellent]; past unstable housing [0=No, 1=Yes]; and HIV disclosure status [0=to none; 1=to someone]). Loneliness mediated the association between stigma and depressive symptoms (β=0.79, SE=0.23, p < .001). The model reflected a very good fit (χ2=0.09, p=.765; CFI=1.00, TLI=1.09, RMSEA < 0.001) and explained 27% of the variance in loneliness and 33% of the variance in depressive symptoms. Stigma predicted higher loneliness, which in turn predicted more depressive symptoms. Findings suggest that addressing depressive symptoms in OPLWH may require multifaceted interventions targeting psychosocial and interpersonal factors including stigma and loneliness.

